# Design, fabrication, and packaging of an integrated, wirelessly-powered optrode array for optogenetics application

**DOI:** 10.3389/fnsys.2015.00069

**Published:** 2015-05-06

**Authors:** Ki Yong Kwon, Hyung-Min Lee, Maysam Ghovanloo, Arthur Weber, Wen Li

**Affiliations:** ^1^Department of Electrical and Computer Engineering, Michigan State UniversityEast Lansing, MI, USA; ^2^School of Electrical and Computer Engineering, Georgia Institute of TechnologyAtlanta, GA, USA; ^3^Department of Physiology, Michigan State UniversityEast Lansing, MI, USA

**Keywords:** optrode array, implantable neural interface, optogenetics, microelectromechanical systems, switched-capacitor based stimulators, wireless power transfer

## Abstract

The recent development of optogenetics has created an increased demand for advancing engineering tools for optical modulation of neural circuitry. This paper details the design, fabrication, integration, and packaging procedures of a wirelessly-powered, light emitting diode (LED) coupled optrode neural interface for optogenetic studies. The LED-coupled optrode array employs microscale LED (μLED) chips and polymer-based microwaveguides to deliver light into multi-level cortical networks, coupled with microelectrodes to record spontaneous changes in neural activity. An integrated, implantable, switched-capacitor based stimulator (SCS) system provides high instantaneous power to the μLEDs through an inductive link to emit sufficient light and evoke neural activities. The presented system is mechanically flexible, biocompatible, miniaturized, and lightweight, suitable for chronic implantation in small freely behaving animals. The design of this system is scalable and its manufacturing is cost effective through batch fabrication using microelectromechanical systems (MEMS) technology. It can be adopted by other groups and customized for specific needs of individual experiments.

## Introduction

Optogenetics is an emerging technique that combines optical and genetic tools for light modulation of neural activity. A key advantage of optogenetics in neuroscience is the ability to excite or inhibit specific cell types with millisecond precision and rapid reversibility (Boyden et al., 2005; Zhang et al., 2007; Deisseroth, 2011; Gerits and Vanduffel, 2013; Zalocusky and Deisseroth, 2013). Recently, neural transfection with substantially red-shifted channelrhodopsin or halorhodopsin genes has demonstrated the potential for deep transcranial optogenetic excitation and inhibition non-invasively (Zhang et al., 2008; Lin et al., 2013; Chuong et al., 2014). Recent emphasis on use of optogenetics has created an increased demand for optical devices targeting delivery of light not only to the surface, but also into deeper subregions of the brain. Examples of surface light delivery systems are acousto-optic deflectors (Losavio et al., 2011), digital micro-mirror devices (DMDs; Arrenberg et al., 2010; Zhu et al., 2012), and computer-generated holograms (CGHs; Reutsky-Gefen et al., 2013). To deliver light to deeper regions of neural tissue, laser- or light-emitting-diode (LED) coupled optical fibers were initially implanted within the brain (Aravanis et al., 2007; Han et al., 2009; Zhang et al., 2010). Recently, more advanced methods, based on micro-/nanofabrication technologies, have been developed to increase the spatial resolution of optogenetic light stimulation of the three-dimensional neural networks of the brain. Such devices include, multichannel silicon oxynitride waveguide arrays (Zorzos et al., 2012), arrayed optical fibers (Royer et al., 2010; Stark et al., 2012), multi-waveguides fabricated on a single substrate (Zorzos et al., 2010), multipoint-emitting optical fibers (Pisanello et al., 2014), optrodes (Wang et al., 2012; Wu et al., 2013), and polymer-based flexible micro-LEDs arrays (Kim et al., 2013).

Despite the aforementioned development of light delivery devices, only limited methods currently are available for use in experiments with freely behaving subjects. These include, laser- or LED-coupled optical fibers, a polymer-based micro-LED array (Kim et al., 2013), and a head-mountable LED system (Iwai et al., 2011). The poor spatial resolution of such systems limits their functionality, and the tethered optical fiber greatly restricts the natural behaviors of the subjects. In addition, since most optogenetic neural probes integrate rigid silica fibers, their mechanical reliability is compromised by optoelectronic integration. As a solution to the current demand for multichannel, bi-directional optogenetic tools, our group has developed a series of flexible, hybrid neural interfacing devices for simultaneous optical modulation and electrical recording of neural activity at multiple levels of cortex. Depending on the interface location, three different devices are available: an Opto-μECoG array for epidural stimulation (Kwon et al., 2012, 2013c), a three dimension (3-D) waveguide array for deep cortical stimulation (Kwon and Li, 2013), and a slanted 3-D waveguide array for multi cortical layers stimulation (Kwon et al., 2013b). Such devices are microfabricated using Parylene-C as the flexible substrate and as the packaging material, thus ensuring biocompatibility while maintaining mechanical compliance (Takeuchi et al., 2005; Rodger et al., 2008; Li et al., 2010, 2011). Using microscale LEDs (μLEDs) as light sources provides several unique advantages compared to the use of external lasers and/or array-based diode lasers, including low power consumption, illumination stability, and fast light switching ability (Mohanty and Thakor, 2013). Integration of individually addressable μLEDs with microwaveguides allows for precise light delivery to the target neurons in individual cortical layers. Furthermore, electronically driven LEDs are particularly suitable for integration with wireless telemetry systems.

Building on the device development, most recently we have implemented a wireless neural interface system. Unlike standard optogenetic approaches that rely on rigid fiber optics tethered to external light sources, our system integrates a microfabricated LED-coupled optrode array with a switched-capacitor stimulator (SCS) and wireless telemetry on a single polymer platform toward a truly untethered bi-directional neural interface for optogenetic application (Kwon et al., 2014a; Lee et al., 2014). This system is flexible, miniaturized and lightweight, and thus suitable for use in small freely behaving animals. The design of our system is scalable and its manufacturing is cost effective, owing to the advantages of batch microfabrication technologies. In this paper, we present the detailed design principle and microfabrication protocols of the slanted LED-coupled optrode array, integration and packaging processes with the wireless system, and demonstration of *in vivo* neural recording and stimulation in rats. The methods described here provide a completely customizable approach for other groups and researchers to design and implement devices for specific needs of individual experiments.

## Materials and Methods

### Design of the Proposed System

**Figure 1:**
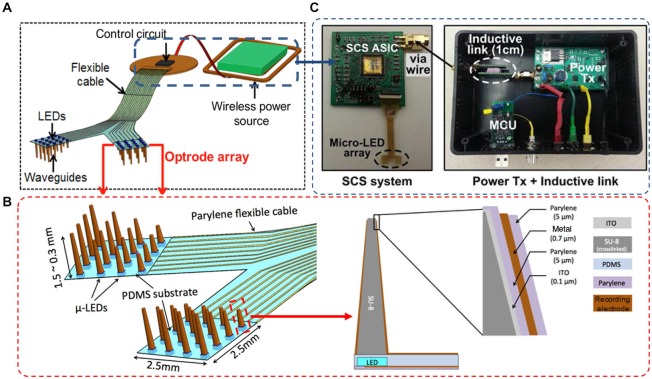
**(A)** Conceptual illustration of the wireless optrode neural interface. **(B)** Overall design of the LED-coupled optrode array with 32 μLEDs, microwaveguides, and microelectrodes integrated on a Parylene-C substrate. **(C)** The inductively-powered telemetry and switched-capacitor based stimulating (SCS) system used to wirelessly drive the μLED array.

As depicted in Figure 1, our wireless neural interfacing system contains three key components: a LED-coupled slanted optrode array, a wireless switched-capacitor based stimulator (SCS), and an inductive link for power transfer. The array has 32 embedded μLED light sources, with 4 × 4 channels per hemisphere on a 2.5 × 2.5 mm^2^ flexible substrate to cover the rat visual cortices bilaterally. The μLED chips are coupled to slanted SU-8 microwaveguide cores, fabricated separately on a polydimethylsiloxane (PDMS) substrate. The design of the needle-shaped waveguide is simulated using a numerical ray-tracing method in TracePro (TracePro®, Lambda Research Co., MA, USA), as reported previously in Kwon et al. (2014b). The simulation results suggest that, given the same base size, the illumination range of the optrode is determined by the tip size of the waveguide core. In particular, a small tip results in divergent irradiance and low output light intensity due to the secondary reflection of light beams along the sidewalls of the tapered waveguide. Optrode with a large tip will enable high irradiance with a confined beam shape. For example, the radius of the illumination area increases from ~0.2 mm to ~0.6 mm when the tip diameter decreases from 100 μm to 10 μm. On the other hand, a large tip may cause more damage to brain tissue during implantation. Based on the simulation results, we designed the needle-shaped waveguides with various lengths ranging from 300–1500 μm in order to target neurons located in different cortical layers, a tip diameter of 30 μm to facilitate device implantation, and a base diameter of ~300 μm to match the dimensions of the μLED chip (220 μm (W) × 270 μm (L) × 50 μm (H)). The cladding layer provided with each individual microwaveguide makes it possible to record electrophysiological signals in response to optical stimulation while preventing light leakage through the sidewalls of the tapered microneedles. To minimize the light-induced artifacts during recording, the cladding layer of the waveguide is specifically designed with four layers of an oxide-polymer-metal-polymer sandwich structure, as depicted in Figure 1B. Starting from the inner layer, transparent indium tin oxide (ITO) is used as the electrical shielding layer to eliminate photoelectrical artifact without compromising light throughput. After an insulating layer of Parylene-C, an opaque metallic layer is constructed to block out light side-leakage, with the tip being etched to allow light delivery to adjacent neurons expressing optogenetic opsins. This layer also is used for constructing the recording electrodes and leads to interconnect with the peripheral elements. The outermost layer is made of Parylene-C for encapsulation. The tip is selectively etched to expose the electrode side for neural recording.

The optrode array is inductively powered and controlled by a wireless switched-capacitor based stimulation (SCS) system, designed at Georgia Institute of Technology to improve power efficiency over traditional current-controlled stimulators, while being capable of delivering instantaneous high power to the μLEDs (Lee et al., 2013). During optical stimulation, the SCS system charges a small array of storage capacitors directly from the inductive link and periodically discharges them into the μLED array at the stimulation onsets, providing high instantaneous current without burdening the inductive link and system supply. To control stimulation parameters, a custom-designed PC interface wirelessly sends data to the SCS system through the inductive link, while a commercial neural recording system simultaneously detects the evoked neural signals from the same optrode array. For a chronically implantable system, all electronic components can be assembled on a mechanically flexible printed circuit board (PCB) substrate and encapsulated with Parylene-C to improve biocompatibility of the system by protecting the electronics from the corrosive biological environment. We graphically designed the masks of the optrode array and the PCB substrate in the AutoCAD environment (Autodesk Inc.), constructed the optrode array using polymer-based microelectromechanical systems (MEMS) techniques, and assembled the components on the PCB substrate using a customized assembly method. Details of the microfabrication and integration are described in the following sections.

### Microfabrication Methods

To reduce fabrication complexity and improve the production yield, the multi-LED array and the slanted optrode array are fabricated and calibrated separately, then bonded together using SU-8 adhesive.

#### Fabrication of the LED Array

A process flow for making the multi-LED array has been developed previously in Kwon et al. (2013c), based on microfabrication and self-assembly techniques. (a-1) A 3-inch glass carrying wafer was cleaned by sonication in acetone, isopropyl alcohol (IPA), and de-ionized (DI) water (3 mins each), followed by Parylene-C deposition (5 μm thick) in a chemical vapor deposition (CVD) system (PDS2010 Parylene Coater, Specialty Coating System, IN, USA). (a-2) A 0.3 μm copper layer was deposited in a thermal evaporator (Edward Auto306, Edwards, UK) and patterned by wet etching to form contact pads and interconnection leads. A photoresist (PR) mask was selectively patterned to expose only the metal contacts for μLED connections. Copper was used to prove the concept, but can be replaced with gold or other conductive materials to improve chemical resistance and biocompatibility. (a-3) Low melting point (LMP) solder (melting point ~62°C, 144 ALLOY Field’s Metal, Rotometals, Inc., CA, USA) was applied on the contacts. The substrate then was rinsed with acetone, IPA, and DI water to remove the PR layer. (a-4) A PDMS (Sylgard 184, Dow Corning, MI, USA) stamp with the embedded blue μLEDs (Cree® TR2227TM die-form LED, peak wavelength at 460 nm, Cree, NC, USA)^1^ was aligned to match the pre-soldered receiver sites on the carrying substrate. Different color LED chips (e.g., green, yellow, or red) can be used for optogenetic inhibition or excitation. (a-5) The substrate with the aligned PDMS stamp was heated on a hot plate at 90°C for 30 s. The PDMS stamp was removed carefully after the substrate had cooled to 40°C, leaving the LEDs attached to the contact pads of the substrate. The substrate then was submerged into an acidic water bath (90°C, pH of 2.0) for 1 min. This step permits fine adjustment of LED alignment and the formation of an electrical connection in a self-assembly manner, utilizing the surface tension of the LMP solder.

The PDMS stamp facilitates the assembly of multiple LEDs on the carrying substrate in a precise and time-efficient way. To fabricate the PDMS stamp, (b-1) a 3-inch glass wafer was first cleaned and went through a dehydration bake. A ~30 μm SU-8 layer was spun onto the wafer and patterned as the mold for fabricating a PDMS stamp. (b-2) PDMS was poured over the SU-8 mold to form the stamp, which contained cavities matching the shape of the μLED chip. (b-3) After curing the PDMS for 40 min on a 95°C hotplate, the stamp was peeled off the mold. (b-4) Thirty-two LED chips were aligned in the cavities of the stamp, with metal pads facing outward. The assembly method using the PDMS stamp was adapted from Onoe et al. (2009), and the yield of the chip assembly was above 96%.

#### Fabrication of the Optrode Array and Assembly

**Figure 2:**
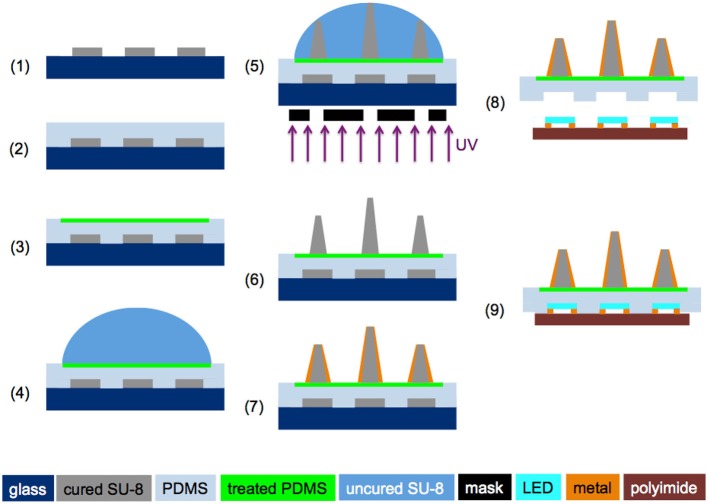
**Fabrication process flow for making the slanted optrode array (steps 1–7) and microassembly of the optrode array with the LED array (steps 8 and 9)**. ©[2014] IEEE. Reprinted, with permission, from Kwon et al. (2014a).

As described in Figure 2, (1) a 3-inch glass wafer was cleaned and put through a dehydration bake, and a ~50 μm SU-8 (SU-8 3025, MicroChem Corp, MA, USA) layer was spun onto the wafer and patterned as the mock LEDs; (2) A thin layer of PDMS was spun onto the SU-8 master to create cavities matching the shape of the LED; (3) After the PDMS was cured for 40 min at 95°C, PR was patterned on the PDMS substrate to expose 7 mm-diameter circles, followed by oxygen plasma treatment to convert the exposed hydrophobic areas to hydrophilic ones; (4) After removal of the PR mask, ~45 μL SU-8 (SU-8 3005) was dispensed on top of the plasma treated PDMS surface using a micropipette; and (5) patterned with a backside droplet backside exposure (DBE) method that utilizes the height variance in a dome-shaped SU-8 structure to create out-of-plane microneedles of varying lengths (Kwon et al., 2013a, 2014b); (6) After SU-8 development, the array was polished by O_2_ plasma etching; (7) DC sputtering of a 0.1-μm-thick ITO layer was performed in a Kurt Lesker Axiss PVD System, followed by deposition of 5 μm Parylene-C. Then a 0.2-μm-thick Au layer was deposited in the thermal evaporator, and the tip of the waveguide was chemically etched for light delivery, followed by deposition of 5 μm Parylene-C as a protective layer. The Parylene-C film at the tip of the optrode was removed using reactive-ion etching (RIE); and (8) The optrode array then was released from the glass wafer, and the microneedles with the matched LED cavities were aligned onto the corresponding LEDs. Finally, (9) the LED array and optrode array were bonded together, with a ~50 μm SU-8 (SU-8 3025) layer spun on the LED array as an adhesive.

### System Integration and Packaging

#### SCS System and Inductive Telemetry

**Figure 3:**
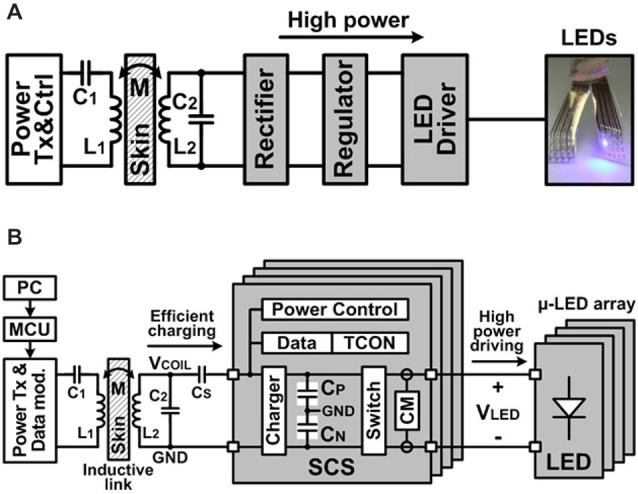
**(A)** Simplified block diagram of a conventional inductively-powered device combined with an array of LEDs for wireless optogenetics and **(B)** simplified block diagram of the wireless switched-capacitor based stimulating (SCS) system to efficiently drive the μLED-coupled optrode array. ©[2014] IEEE. Reprinted, with permission, from Lee et al. (2014).

Integrated LED light sources powered by the wireless SCS system enable a truly untethered optogenetic stimulation system. Inductive power transmission across the skin has been a viable solution to provide sufficient power to the implantable medical devices (IMDs), while overcoming size, cost, and longevity constraints of embedded primary batteries. Light sources in optogenetics, however, typically require high instantaneous power to emit sufficient light for optical neural stimulation, which can be a significant limiting factor in conventional IMDs (Wentz et al., 2011). Figure 3A shows the conceptual block diagram of a conventional inductively-powered array of LEDs for wireless optogenetics, where a rectifier and a regulator convert AC voltage across a secondary coil, *L_2_*, to DC output voltage to supply a LED driver. Power losses in these stages result in poor overall power efficiency from *L_2_* to the LED. Moreover, high instantaneous power that flows to the LEDs when they are on, leads to large load variation, which affects the impedance matching with the inductive link. This will significantly increase the required inductive power level, creating safety issues, and degrading the inductive link power efficiency as well as supply voltage for the rest of the IMD. To address these limitations in implantable optogenetics stimulators, the SCS system has been designed and implemented for power-efficient wireless optical stimulation, as shown in Figure 3B. The proposed SCS system efficiently charges a bank of storage capacitors, *C_S_*, directly from *V_COIL_* through the inductive link without using any rectifiers or regulators, improving the capacitor charging efficiency. Moreover, the charge stored in capacitors is delivered to the load through large switches, efficiently creating high current stimulation pulses.

#### Integration and Hermetic Package for Chronic Implants

**Figure 4:**
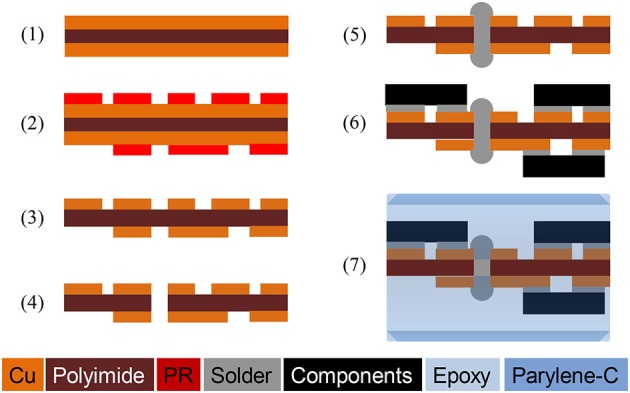
**Fabrication process of a hermetically-sealed chronic implant on a flexible polyimide circuit**. ©[2014] IEEE. Reprinted, with permission, from Lee et al. (2014).

Figure 4 illustrates the process flow for integration and hermetic packaging of a chronic implant on a flexible polyimide PCB substrate for experiments using freely behaving animals. The flexible polyimide PCB substrate was constructed using Pyralux®AP (AP7163E, DuPont) with the following steps: (1) A 3-inch Pyralux® wafer was cut and cleaned, and a 3-μm thick PR layer was spin coated; (2) The circuit design was transferred on to the PR using a lithography technique; (3) The circuit was patterned by wet etching of copper; (4) Through holes were made by a laser cutter; (5) Vias were made by filling the through holes with solder; (6) Solder paste was applied on the contact pads and the SCS chip, and other surface mount devices (SMDs) were populated on the pads. The circuit was baked at 200°C for 5 min, and extra flux was applied for reflow soldering, if necessary; and (7) Once all components were populated, a thick layer of medical-grade epoxy (200~500 μm, EP21LVMed, Master Bond) was applied, followed by a 10 μm Parylene-C coating as a biocompatible package.

### *In Vivo* Experiment in Rats

*In vivo* acute animal experiments have been conducted to demonstrate the ability of the LED-coupled optrode array to simultaneously modulate and record neural activities in the primary visual cortex (V1) of a rat. All procedures were approved by the Institutional Animal Care and Use Committee (IACUC) at Michigan State University. Two weeks prior to the experiments, rodent subjects (Sprague-Dawley rats: 250–400 g) had neurons in their V1 transfected with the AAV-hSyn-hChR2(H134R)-mCherry viral vector (10 × 10^11^ ~10 × 10^12^ vector genome (vg)/mL; UNC Vector Core, NC, USA). During the viral injection, each animal was anesthetized with ketamine/xylazine (80 mg/kg: 5 mg/kg i.m.) and anesthesia maintained with ketamine (20 mg/kg i.m.) as needed. The eyes were treated with 1% methylcellulose to prevent corneal drying. Using sterile surgical procedures, a 3–4 cm incision was made in the skin overlying the skull and small holes were drilled through the bone over V1. Using a glass pipette and a pressure injection system, 1.0 μL of the virus solution was injected at 3–4 equidistant locations. Injections occurred over a 2–3 min period, and the glass pipette was left in place for an additional 10 min to allow the vector to diffuse away from the injection site. The cortex then was covered with Gelfoam and the skin overlying the skull was sutured closed. Post-surgery, the animal received pain medication (buprenorphine; 0.05 mg/kg s.c.) and fluids (10–12 cc sterile saline, s.c.) subcutaneously and was placed in a cage on a heating pad until fully recovered, after which the animal was returned to the animal care facility. For device implantation, the animal expressing ChR2 (2–4 weeks after virus injection) was anesthetized using the procedure described above. The anesthetized animal was placed in a stereotaxic apparatus and a small region (4 × 4 mm^2^) of the skull over V1 was removed. The optrode array was surgically implanted over V1, with the LEDs staying on the surface of the cortex and the waveguides inserted in the cortical tissues.

**Figure 5:**
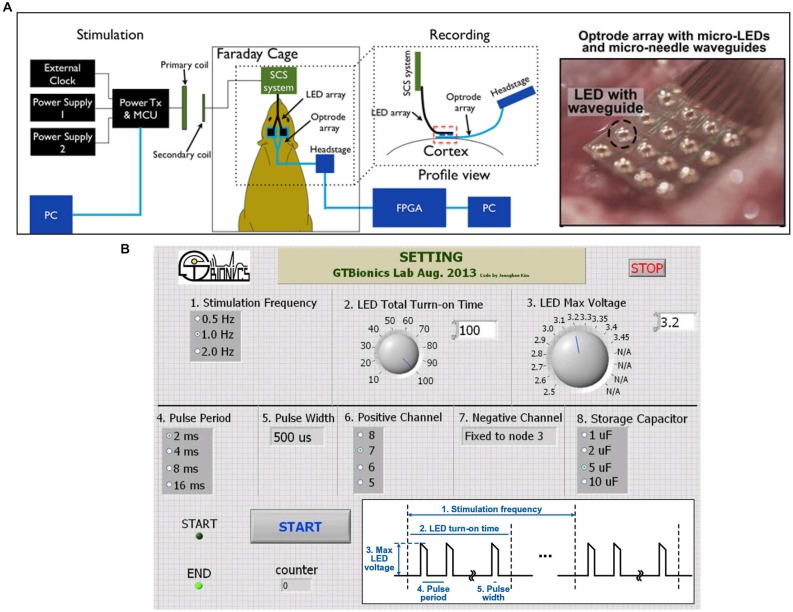
**(A)** *In vivo* animal experiment setup with the wireless SCS system. **(B)** A SCS system GUI in LabVIEW to wirelessly control the SCS parameters. Inset shows the LED driving voltage waveform and its controllable parameters.

After device implantation, acute experiments were conducted in one transfected animal to validate the functionality of the implanted device. The experimental setup is shown in Figure 5A, where the SCS system was used to power and control the 32-channel LED array and a 32-channel headstage (Intan RHD2132) was used to receive and process the neural signals recorded through the optrode. A Faraday cage was used to isolate the animal, the optrode array, the integrated SCS system, and the recording headstage from the environmental interference in order to improve the signal-to-noise ratio of the neural recordings. The SCS system receives wireless power and data through the inductive link and drives μLEDs in the optrode array. The proof-of-concept prototype of the SCS system occupies 3.9 × 3.9 cm^2^ on the PCB, and includes off-chip components for testing and optrode connectors. Key components of this prototype are the SCS chip (5 × 2.4 mm^2^) and four pairs of 1~5 μF off-chip storage capacitors (SMD-0402, 1 × 0.5 mm^2^ each). The SCS system can be miniaturized further using a single flex-PCB method described previously in Figure 4, which can connect directly to the LED array and include the receiver coil, *L_2_*, without connectors or testing components. Storage capacitors can be placed on the opposite side of the SCS chip, minimizing the PCB size for chronically implantable optogenetics. Neural signals were recorded simultaneously from the penetrating electrodes on the same optrode array through an Intan RHD2132 evaluation board and its commercial hardwired recording setup (Intan Technologies, CA, USA).

To selectively turn on the LEDs and facilitate the adjustment of stimulation parameters, a graphical user interface (GUI) has been implemented in the LabVIEW environment (National Instruments, Austin, TX) to send data packets from the PC to the microcontroller unit (MCU) in the power transmitter (Tx) module, as shown in Figure 5B. Several parameters of the LED driving signal can be adjusted in this setup: (1) stimulation frequency, 0.5~2 Hz; (2) LED total turn-on time, 10~100 ms; (3) LED peak voltage, 2.5~3.45 V; (4) pulse period, 2~16 ms; (5) pulse width, 512 μs; (6) positive output connections, 4 channels; (7) negative output connections, fixed; and (8) storage capacitance, 1~10 μF. While the pulse train periodically turns the LEDs on and off, the effective turn-on time can be simply calculated as (total turn-on time) × (pulse width/pulse period).

## Results and Discussions

### Fabrication Results

**Figure 6:**
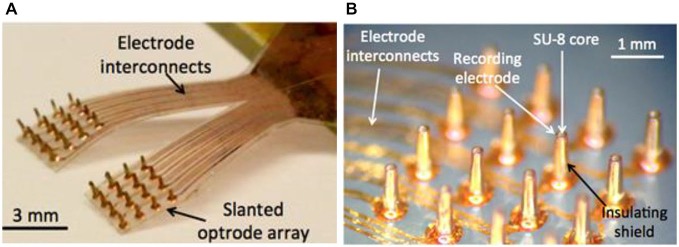
**Fabricated prototypes of (A) a 32-channel LED-coupled optrode array; and (B) microscopic image of individual optrodes**.

**Figure 7:**
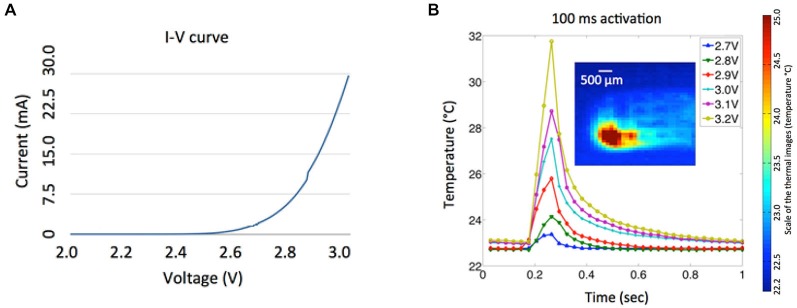
**(A)** Measured I-V characteristic of a μLED chip. **(B)** Temperature variances of the LED array at various input voltages (2.7–3.2 V) for 100 ms activations. The inset shows the corresponding thermal image of a single LED activation taken at the maximum temperature with 3.2 V input voltage and activation duration of 100 ms.

Figure 6 shows the photo images of a LED-coupled optrode array. The electrode-electrolyte interface impedances of the optrode array were measured at 1 kHz using a built-in electrode-impedance-testing circuitry in an Intan evaluation board (RHD2132 and RHD2000-EVAL, Intan Tech. LLC, CA, USA). The impedances of the recording electrodes ranged from 10–500 kΩ, suitable for local field potential (LFP) recordings. The impedance of the optrode can be adjusted by controlling the size of the Parylene-C opening, using the process in Figures 2–7.

Power consumption of the μLED at various operating conditions was investigated (Figure 7A) to provide useful guidelines for optical stimulation in *in vivo* experiments and future integration of the LED array with wireless power telemetry. The nonlinear current-voltage characteristic of a single μLED was measured with an impedance analyzer (HP 4191A RF, Hewlett Packard, CA, USA). Since a minimum light-energy density of 1 mW/mm^2^ must be delivered onto the target area to effectively excite channelrhodopsin-2 (ChR2; Grossman et al., 2010), a minimum input voltage of ~3.2 V is required to drive a single μLED, resulting in electrical power consumption of 27 mW and average light intensity of 1.4 mW/mm^2^ from the waveguide tip. The temperature variation of the LED array was measured using a high definition infrared camera (Delta Therm. HS1570 and its software (DT v.2.19)) with different input voltages (2.7–3.2 V) and activation duration of 100 ms, as shown in Figure 7B. Thermal images were taken in air under an environment (at room temperature of 22.7°C) where ventilation was strictly controlled to minimize ambient thermal noise, and the array was suspended in the air during the LED activation. As expected, the localized temperature of the LED increases dramatically as the increase of the input voltage. The relatively high input voltage of the LED (3.2 V) can be attributed to inefficient light coupling between the LED chip and the tapered microwaveguide, which typically has a coupling efficiency of ~10% for the butt coupling (Keiser, 2008). It is of note that, in Figure 7B, the pulse train of 3.2 V and 100 ms resulted in the temperature rise by more than ~10°C, which may affect neural function and even cause tissue damage. However, this experiment was carried out in air without the thermoregulation effect of the cerebral circulation. To evaluate the safety of our simulation paradigm, we measured the temperature variation of the cortical region close to the implantation site using a thermocouple, when the LED was driven by a continuous train of 3.2 V, 100 ms pulse width, and 2 Hz frequency. Preliminary results show that the maximal temperature rise was approximately 0.1–0.4°C, which satisfies the safety requirement for implantable neuroprosthetics. Comprehensive investigation will be performed in the future to fully characterize the thermal properties of the integrated system.

To evaluate the performance of epoxy-Parylene-C packages and the possible electrical failure of the chronic implant, active accelerated-lifetime soak testing was performed in saline (a solution of 0.90% w/v of NaCl) at a higher than body temperature. For soak testing, a discrete version of the SCS circuit was designed using a MCU (MSP430, Texas Instruments, TX, USA), and constructed using the process described in Figure 4. This circuit was programmed to mimic SCS stimulation patterns, once it received wireless power and data through the inductive link. Dimensions and fabrication process of the discrete circuit were identical to the SCS system. Four LEDs (LB QH9G, blue 466 nm, OSRAM, Germany) were integrated on the flexible PCB, and each LED was individually controlled by the MCU. A MEMS-based receiver (Rx) coil was fabricated separately to be placed on the back of the animal and connected to the SCS with flexible wires (392 F high-flex miniature wire, 36 AWG, McMaster-Carr. OH, USA).

**Figure 8:**
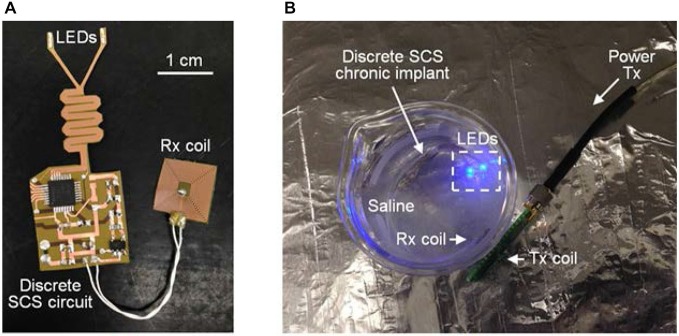
**(A)** Discrete SCS chronic implant and **(B)** accelerated soak testing in saline with wireless power and data transfer. ©[2014] IEEE. Reprinted, with permission, from Lee et al. (2014).

Five discrete SCS devices were prepared and immersed in saline at 75°C, as shown in Figure 8B. Each individual device was activated by coupling the Rx and Tx coils, and daily the samples were visually monitored for possible device failure. No delamination, major physical corrosion, or performance degradation occurred after 14 days in accelerated lifetime testing conditions. Using the Arrhenius relationship, preliminary results show that the implant lifetime can be the equivalent of 3.5 years at body temperature of 37°C, sufficient for one-year duration requirement in the animal study. Further improvements can be achieved by optimizing temperature and duration of heat treatment for the Parylene/metal thin-film skin or by using additional chemical treatments.

### *In Vivo* Experimental Results

**Figure 9:**
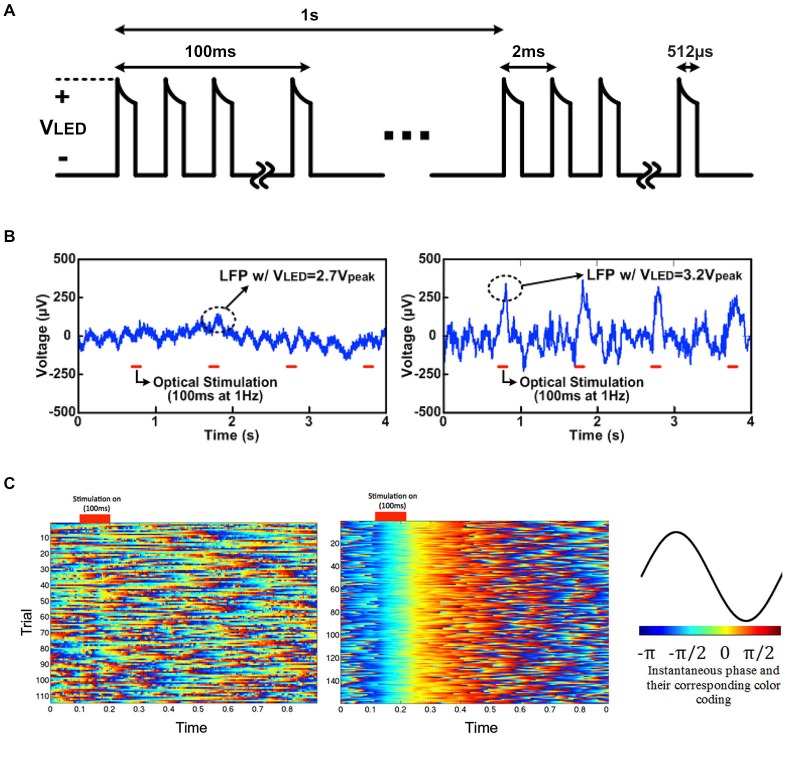
**Light-induced LFP recording driven by 2.7 V_peak_ and 3.2 V_peak_ for 100 ms: (A) LED driving voltage, *V*_LED_, for *in vivo* optogenetics with SCS, (B) light-induced local field potentials (LFP) with *V*_LED_ = 2.7 V_peak_ and 3.2 V_peak_, and (C) instantaneous phase with *V*_LED_ = 2.7 V_peak_ (top) and 3.2 V_peak_ (bottom) in low frequency band (1–25 Hz), and their corresponding color-coding**.

Figure 9 shows the LED driving voltage waveforms, *V_LED_*, for optical stimulation with SCS as well as light-induced *in vivo* LFP results. The LFPs below 500 Hz were recorded using the optrode array with microwaveguides in the rat’s V1 when the SCS system drove μLEDs with a 512 μs pulse train for 100 ms at 1 Hz and *V_LED_* = 2.7 V_peak_ and 3.2 V_peak_, as shown in Figure 9A. For each *V_LED_*, in total 150 trials of optical stimulation and simultaneous recording were performed. A clear and stable light-induced neural activity was observed in LFPs (1–500 Hz) in time domain driven by 3.2 V_peak_ input voltage (Figure 9B). The high input voltage of 3.2 V_peak_ results in an average irradiance of 1.4 mW/mm^2^ at the tip of the waveguide, which is above the minimal irradiance (1 mW/mm^2^) required for light-evoked neural responses. Lower input voltage (for example, 2.7 V_peak_ resulting in the average irradiance of 0.35 mW/mm^2^) was also tried, but no significant neural modulation was observed.

To clearly visualize neural oscillations induced by the optrode array, instantaneous phases of the two datasets (with 2.7 V_peak_ and 3.2 V_peak_ input voltages) of light-evoked LFPs (1–25 Hz) were measured based on Hilbert transform. Each trial was truncated to a ~100 ms pre-stimulation period and an ~800 ms post-stimulation period. The instantaneous phases of each individual trial (1 s with 100 ms light stimulation), labeled with different colors according to Figure 9C, were aligned based on the stimulus ON time and stacked. With 3.2 V_peak_ input voltage, light-evoked neural activity shows strong phase synchronization, while no phase synchrony was observed in neural recording data with 2.7 V_peak_ input voltage. The neural modulation and recording was extremely reliable across over 100 trials during the *in vivo* experiments. The cortically optical stimulation generated almost deterministic phase-locked neuronal oscillations without any latency for ~0.3 s as validated with a nonparametric test (*p* < 0.05, Wilcoxon signed rank test for each pair of channels). This experiment, while still preliminary, has verified the feasibility of wirelessly powered optical stimulation and simultaneous recording of neural activity via the LED-coupled optrode array.

### Discussion and Outlook

Table 1 compares the presented LED-coupled optrode array with some microfabricated, laser-coupled optical/electrical implants reported by other group. As can be seen, our LED-coupled optrode array provides several unique advantages over laser-coupled devices. First, the devices are manufactured using advanced microfabrication techniques, therefore enabling high channel count and fine spatial resolution for optical stimulation and electrical recording. The design of our system is scalable and the developed manufacturing method is cost effective and reliable. In addition, In addition, our approach uses commercially available LED chips coupled to microfabricated SU-8 waveguides, which eliminates the need for tethered optical fibers and thus is suitable for integration with wireless power and data telemetries. While this work is primarily focused on the neural excitation, the wide selection of the LED chips makes it possible to switch the colors of the LED chips to yellow or red for applications in optogenetic neural inhibition.

**Table 1:** **Comparison between our LED-coupled optrode array and other laser-coupled optical/electrical devices**.

Optical neurostimulation			Electrical recording		Wireless-enabled	Ref
Light source	^#^of waveguides	Fiber size	^#^of electrodes	Electrode size
Laser	4 or 8	Tip size: 5~20 μm	8 per probe	~160 μm^2^	No	Royer et al. (2010)
Laser	1	50~100 μm	36	50~100 μm	No	Wang et al. (2012)
Laser	1	Tip size: 14~28 μm	8	20 μm × 20 μm	No	Wu et al. (2013)
Laser	7	~400 μm	16	N.A.	No	Pisanello et al. (2014)
LED	32	Base size: 300 μm Tip size: ~30 μm	32	~30 μm	Yes	Our Optrode Array

For chronic implantation the most critical aspects are certainly the aging behavior of the device and the stability of neural recordings. We anticipate that the performance degradation may be caused by two factors: failure of Parylene-C package and aging of SU-8 waveguide cores. The biocompatible package of the current prototype used 10 μm Parylene-C, which has lower moisture permeability over PDMS and polyimide, and is suitable for packaging of chronic neural implants (Loeb et al., 1977; Schmidt et al., 1988; Takeuchi et al., 2005; Rodger et al., 2008). SU-8 for constructing waveguides on implantable neural probes brings along advantages such as flexibility, optical clarity, and compatibility with conventional photolithography techniques. However, since SU-8 is a photosensitive resist, continuous exposure to blue light may cause the degradation of the material flexibility and optical transparency due to cross-linking of the resin (Fiedler et al., 2014), which will lead to increased coupling loss of light along the waveguides. In this paper, the accelerated short-term soak testing of the optrode prototype has shown good reliability and stability of the Parylene-C package. Although we have not observed any performance degradation of the device in short-term experiments, further long-term studies have to be performed to fully characterize the reliability and stability of the optrode array. More detailed investigation is necessary to optimize the fabrication processes, where, for example, the influence of PR exposure and hard baking should be examined carefully.

## Conclusions

In conclusion, design, fabrication, and testing procedures of the wireless LED-coupled slanted optrode array for a really untethered bi-directional neural interface were presented. The array was inductively powered and controlled by the wireless SCS system, designed to improve power efficiency. The SCS system for implantable wireless optogenetics provides high instantaneous power through the inductive link to emit sufficient light and evoke neural activities. The LabVIEW PC interface and custom-designed power Tx module provide wireless power and data to the SCS system, while electrodes embedded in the optrode array enable simultaneous neural recording. The self-assembled LED array on a single substrate can reduce manufacturing cost. Acute *in vivo* experiments with optical stimulation and LFP recording have verified the efficacy of our system for wireless optogenetics application.

## Conflict of Interest Statement

Wen Li and Ki Yong Kwon, “Neural Prosthetic Device and Method of Making Same,” Pending U.S. Provisional Patent, Application Serial No. 61/845,106, MSU Ref. No. TEC2013-0092-01Prov -SLW Ref. No. 3000.138PRV, 2014. The other authors declare that the research was conducted in the absence of any commercial or financial relationships that could be construed as a potential conflict of interest.

## Footnotes

^1^Cree LED Datasheet. http://www.cree.com/~/media/Files/Cree/LED%20Components%20and%20Modules/XLamp/Data%20and%20Binning/XLampMCE_BL.pdf
